# Fine spatiotemporal activity in contracting myometrium revealed by motion-corrected calcium imaging

**DOI:** 10.1113/jphysiol.2014.275412

**Published:** 2014-08-01

**Authors:** Fiona C Loftus, Anatoly Shmygol, Magnus J E Richardson

**Affiliations:** 1 Warwick Systems Biology Centre, University of WarwickCoventry, UK; 2 Division of Translational and Systems Medicine, Warwick Medical School, University of WarwickCoventry, UK; 3 Warwick Systems Biology Doctoral Training Centre, University of WarwickCoventry, UK

## Abstract

Successful childbirth depends on the occurrence of precisely coordinated uterine contractions during labour. Calcium indicator fluorescence imaging is one of the main techniques for investigating the mechanisms governing this physiological process and its pathologies. The effective spatiotemporal resolution of calcium signals is, however, limited by the motion of contracting tissue: structures of interest in the order of microns can move over a hundred times their width during a contraction. The simultaneous changes in local intensity and tissue configuration make motion tracking a non-trivial problem in image analysis and confound many of the standard techniques. This paper presents a method that tracks local motion throughout the tissue and allows for the almost complete removal of motion artefacts. This provides a stabilized calcium signal down to a pixel resolution, which, for the data examined, is in the order of a few microns. As a byproduct of image stabilization, a complete kinematic description of the contraction–relaxation cycle is also obtained. This contains novel information about the mechanical response of the tissue, such as the identification of a characteristic length scale, in the order of 40–50 μm, below which tissue motion is homogeneous. Applied to our data, we illustrate that the method allows for analyses of calcium dynamics in contracting myometrium in unprecedented spatiotemporal detail. Additionally, we use the kinematics of tissue motion to compare calcium signals at the subcellular level and local contractile motion. The computer code used is provided in a freely modifiable form and has potential applicability to *in vivo* calcium imaging of neural tissue, as well as other smooth muscle tissue.

**Key points**

- Calcium indicator fluorescence imaging is a key tool for investigating the physiological mechanisms that initiate and regulate myometrial contractions. However, inhomogeneous tissue motion severely limits the effective spatial resolution of measurable calcium activity.
- We present an image analysis method that locally tracks contractions to produce a complete description of tissue-wide motion. This renders calcium signals measurable at the micrometre scale. Additionally, the method simultaneously extracts kinematics of the motion that will inform biomechanical models of contracting myometrium.
- The method is demonstrated on calcium-imaging datasets of contracting myometrium. Using the method, we observed significant heterogeneity in calcium activity and identified a characteristic length scale of 40–50 μm below which tissue motion remains locally homogeneous.
- We provide freely available and modifiable code to process datasets affected by motion artefacts. The method has potential application to *in vivo* neuron imaging, as well as to calcium imaging of other smooth muscle tissue.

## Introduction

During normal labour, uterine contractions are precisely timed and spatially coordinated. The treatment of dysfunctional labour, in which this coordination breaks down, requires an understanding of the physiological mechanisms that initiate and regulate myometrial contractions. As pregnancy progresses, the excitability and connectivity of the constituent myocytes increase, manifesting in the depolarization of the membrane potential of the myocytes (Parkington *et al*. 1999) and an increase in the number of gap junction proteins (Garfield *et al*. 1977, 1978; Miyoshi *et al*. 1996; Miller *et al*. 1989). This transformation provides the conditions necessary for the spontaneous generation of coordinated contractions during labour, but the precise mechanisms underlying this coordinated activity remain unclear. The central role of intracellular calcium in activating the contractile machinery in myometrium has meant that a key method for investigating the underlying physiological mechanisms is *in vitro* calcium indicator fluorescence imaging of contracting strips of myometrium.

Ratiometric calcium-sensitive dyes and force transducers have been used to obtain simultaneous measurements of [Ca^2+^]_i_ transients and force in strips of rat (Taggart *et al*. 1996; Longbottom *et al*. 2000; Noble & Wray, 2002; Jones *et al*. 2004) and human (Word *et al*. 1994; Longbottom *et al*. 2000; Fomin *et al*. 2006) myometrium. In spontaneously contracting tissue, when the coupling between [Ca^2+^]_i_ and force production remains intact, a close correlation exists between the two measurements, with [Ca^2+^]_i_ transients peaking before force (Taggart *et al*. 1996; Noble & Wray, 2002; Jones *et al*. 2004). However, the [Ca^2+^]_i_ signals in these studies reflect an average of tissue-wide [Ca^2+^]_i_ activity; although they contribute to an understanding of the relationship between global [Ca^2+^]_i_ transients and total force production, such measurements do not provide information about [Ca^2+^]_i_ signals at the cellular level or the intercellular communication that results in synchronous contractions. Recently, two studies sought to characterize the spatial and temporal propagation of [Ca^2+^]_i_ transients within slices of rat (Burdyga *et al*. 2009) and human (Bru-Mercier *et al*. 2012) myometrium. Burdyga *et al*. (2009) measured calcium indicator transients from individual cells exhibiting non-propagating oscillations. Temporal characteristics of regional [Ca^2+^]_i_ activity were also examined during periods of global [Ca^2+^]_i_ activity and tissue contraction. However, the associated motion of the contracting tissue prevented the measurement of calcium indicator signals at the cellular and subcellular levels during global activity. In Bru-Mercier *et al*. (2012), the [Ca^2+^]_i_ transients of cells both within the same bundle of myocytes and between two bundles during contraction were compared. The [Ca^2+^]_i_ measurements in this study reflect the averages of calcium indicator fluorescence from both the cell and the neighbouring region of tissue. The choice of cells from which calcium indicator fluorescence measurements could be taken was therefore limited: measurements could be obtained only from cells positioned at least as far as their maximal displacement away from neighbouring cells.

Because the concomitant motion of contracting myometrium severely limits the effective spatial resolution of measured [Ca^2+^]_i_ activity, we considered whether it might be possible to process calcium indicator fluorescence imaging sequences post-experiment to remove motion artefacts. Motion-tracking algorithms have long been applied to biological images, in contexts ranging from single-cell and particle tracking to tissue-level motion tracking (Meijering *et al*. 2012; Chenouard *et al*. 2014). Quantification of deformation of heart tissue has developed from analysis of two-dimensional motion in echocardiograms (Mailloux *et al*. 1987, 1989) to three-dimensional descriptions of motion in echocardiograms (Suffoletto *et al*. 2006) and magnetic resonance (MR) images (Mansi *et al*. 2011). Recently, software has been developed to track and correct for motion artefacts in *in vivo* two-photon calcium indicator imaging of neurons (Greenberg & Kerr, 2009; Tomek *et al*. 2013). Suffoletto *et al*. (2006) used speckle tracking to obtain descriptions of cardiac motion; this technique is commonly used in echocardiography, in which it tracks the motion of stable patterns of natural acoustic markers or ‘speckles’. In Tomek *et al*. (2013), the contrast between intra- and extracellular intensity levels was used to track the motion of cells. The algorithms implemented in all other cases relied on the assumption that the local intensity of the imaged tissue remained constant throughout the image sequences. The application of motion-tracking algorithms to contracting myometrium, however, is complicated by the fact that there are simultaneous changes in image intensity. In a study investigating intra- and intercellular Ca^2+^ waves in the murine large intestine, Hennig *et al*. (2002) applied a motion-correction algorithm to calcium indicator fluorescence images of this contracting smooth muscle tissue. In this case, the imaged tissue contained aligned longitudinal muscle cells, and the direction of motion was therefore similar throughout the imaged slice. To our knowledge, no algorithms have been developed to correct for motion artefacts in calcium indicator fluorescence imaging of highly contracting tissue in which spatially heterogeneous movements occur simultaneously within the field of view.

Here, we present a novel technique that tracks local motion in confocal images of myometrium loaded with calcium-sensitive dye. It removes the motion artefacts to allow for a spatially detailed analysis of [Ca^2+^]_i_ signals and additionally provides a full kinematic description of tissue motion during contraction and relaxation cycles. Firstly, an overview and a detailed description of the method are provided in the Methods section. A modifiable example of the computer code and its application to a dataset are provided in the Supporting Information. We then demonstrate the application of the method in a variety of datasets. We show how the method can be used to measure local calcium indicator signals and how such signals taken from multiple sub-regions of tissue can be analysed for spatiotemporal correlations. In addition to examining the calcium signal, the kinematic description obtained through motion tracking is used to compare the spatiotemporal profiles of local [Ca^2+^]_i_ activity and tissue contraction. The tissue kinematics are also used to examine contractile properties of the tissue slices, which comprise multiple myocyte bundles separated by interstitial space and therefore exhibit significant spatial heterogeneity of motion during a contraction. This includes the identification of a characteristic length scale, in the order of 40–50 μm, below which tissue motion remains locally homogeneous during a contraction.

## Methods

After the experimental data are described, an overview of the method is given for readers more interested in using the software and less concerned with the specifics of implementation. A more detailed account of the method with specifics of the algorithm is then provided. Note that a working example of the code, written in the language of the matlab (The MathWorks, Inc., Natick, MA, USA) environment, together with an example dataset are provided as part of the Supporting Information. These may be freely used and modified.

### Ethical approval

The study conformed to the tenets of the *Declaration of Helsinki* and was approved by the local ethics committee at University Hospital Coventry and Warwickshire (REC-05/Q2802/107). Myometrial biopsy specimens were obtained with informed written consent (information leaflet ref. PTL220705) from term-pregnant women (≥37 weeks gestation) undergoing elective caesarean section before the onset of labour.

### Experimental data

The data used to test the algorithm were obtained using confocal imaging of 200 μm-thick myometrial slices loaded with Fluo-4/AM (Invitrogen Corp., Paisley, UK). Full details are provided in Bru-Mercier *et al*. (2012). Briefly, each biopsy specimen was trimmed into a strip, which was then ligatured at both ends before being stretched and fixed to the base of a stainless steel tissue holder. From this, 200 μm-thick slices were cut. For [Ca^2+^]_i_ recording, the slices were incubated in Krebs solution containing 13 μm Fluo-4/AM. The loaded slice was placed in a glass-bottomed Petri dish and weighted down with a 250 mg slice grid. This was secured on the stage of an inverted microscope equipped with an LSM 510 META confocal scanner and superfused with pre-warmed Krebs solution until stable spontaneous contractions developed. Confocal imaging of Fluo-4/AM loaded slices was achieved by scanning a 488 nm wavelength laser beam focused into a diffraction-limited spot via a Fluar 5×/0.25NA objective lens and recording fluorescence through a bandpass filter (505–530 nm) using a photomultiplier tube with a pinhole in front of it. The frame size of the images ranged between 217 × 412 pixels and 512 × 512 pixels, and pixel width ranged between 1.0 μm and 4.5 μm. The recordings were made at a rate of one or two frames per second (fps) for recording times of 12–67 min. For some of the datasets, the tissue was subject to additional pharmacological protocols, including control conditions, nifedipine (1 μm), oxytocin (10–100 nm) or combinations of these. For the purposes of the present study, the exact nature of the protocols is not relevant; rather, it should be noted that a broad range of [Ca^2+^]_i_ and contractile activity were exhibited in order to test the robustness of the motion-correction algorithm.

### Overview of the motion-correction algorithm

**Figure 1:**
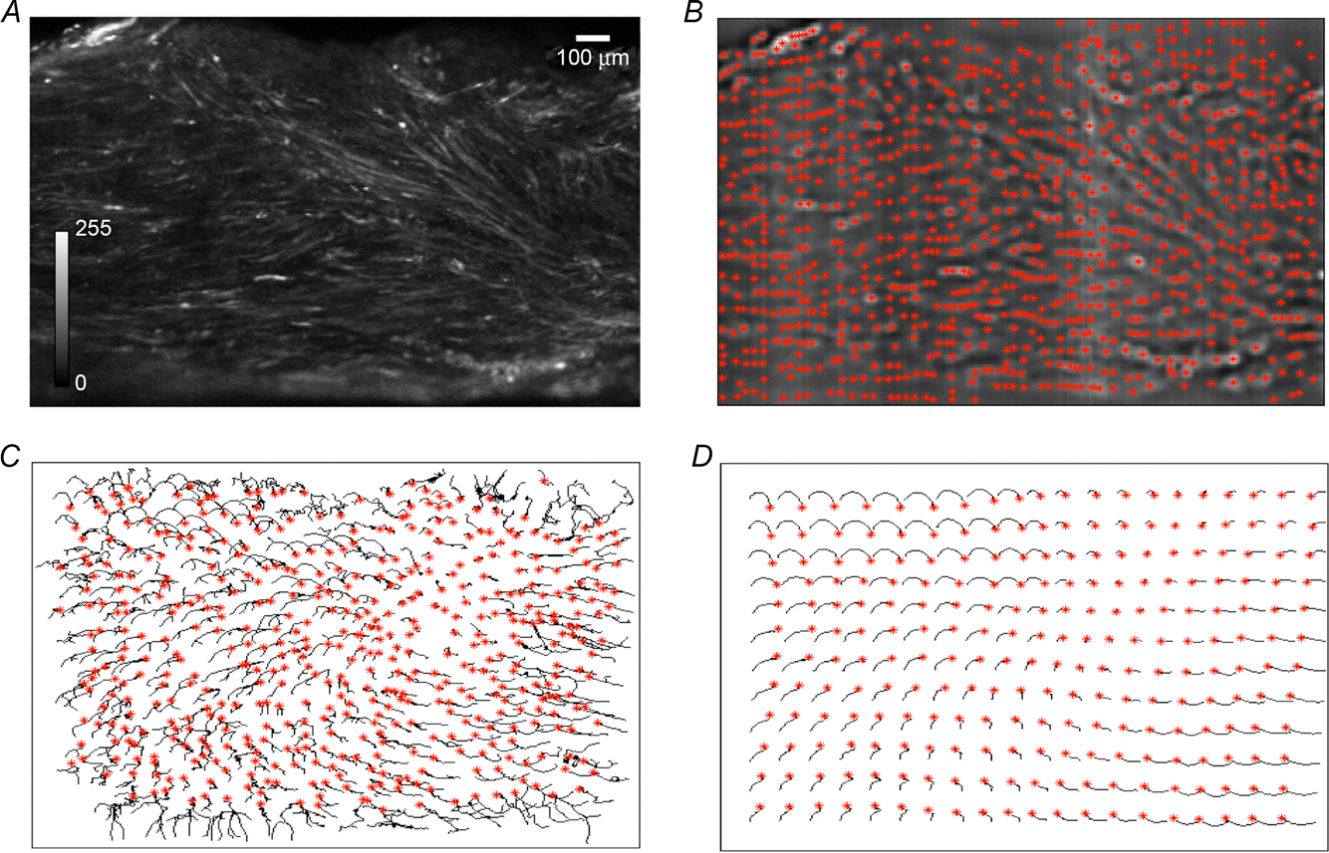
Overview of the motion-correction algorithm *A*, raw fluorescence image of a Fluo-4 loaded myometrial slice. *B*, the raw image is bandpass filtered to emphasize small cell bodies (landmarks) with radii of ∼15 μm. Landmarks suitable for tracking are identified (red*) as those having a high correlation with a two-dimensional Gaussian function. *C*, the landmarks are then tracked by placing a small window over each landmark and looking within the window in the next frame for the displaced landmark. The data show the trajectories during a single contraction with the final frame indicated (red*). The trajectory of each landmark is tested for regularity and any outliers are removed from the dataset. *D*, the motion of the landmarks is then extrapolated to neighbouring pixels to provide a complete description of the tissue-wide motion. The extrapolation procedure also identifies a characteristic length scale below which tissue motion is homogeneous. Note that for clarity tracks in (*D*) are shown only for every 20th pixel (separation of 90 μm).

The main steps of the algorithm are shown in Fig. 1 (see also Supporting Movie 1) and comprise: (i) identifying landmarks by bandpass filtering the image to emphasize small cell bodies of 30 μm in size; (ii) tracking the motion of each landmark between frames for the entire image stack and removing outlier landmarks, and (iii) extrapolating the motion of landmarks to all neighbouring pixels to yield a complete description of the tissue motion. This requires identifying a characteristic length scale below which tissue motion is homogeneous. This tissue motion description constitutes the calcium-signal time series and kinematic data – position, velocity and acceleration as functions of time – for the tissue under each pixel in the initial frame.

### Details of the motion-correction algorithm

#### Identification of landmarks for tracking

**Figure 2:**
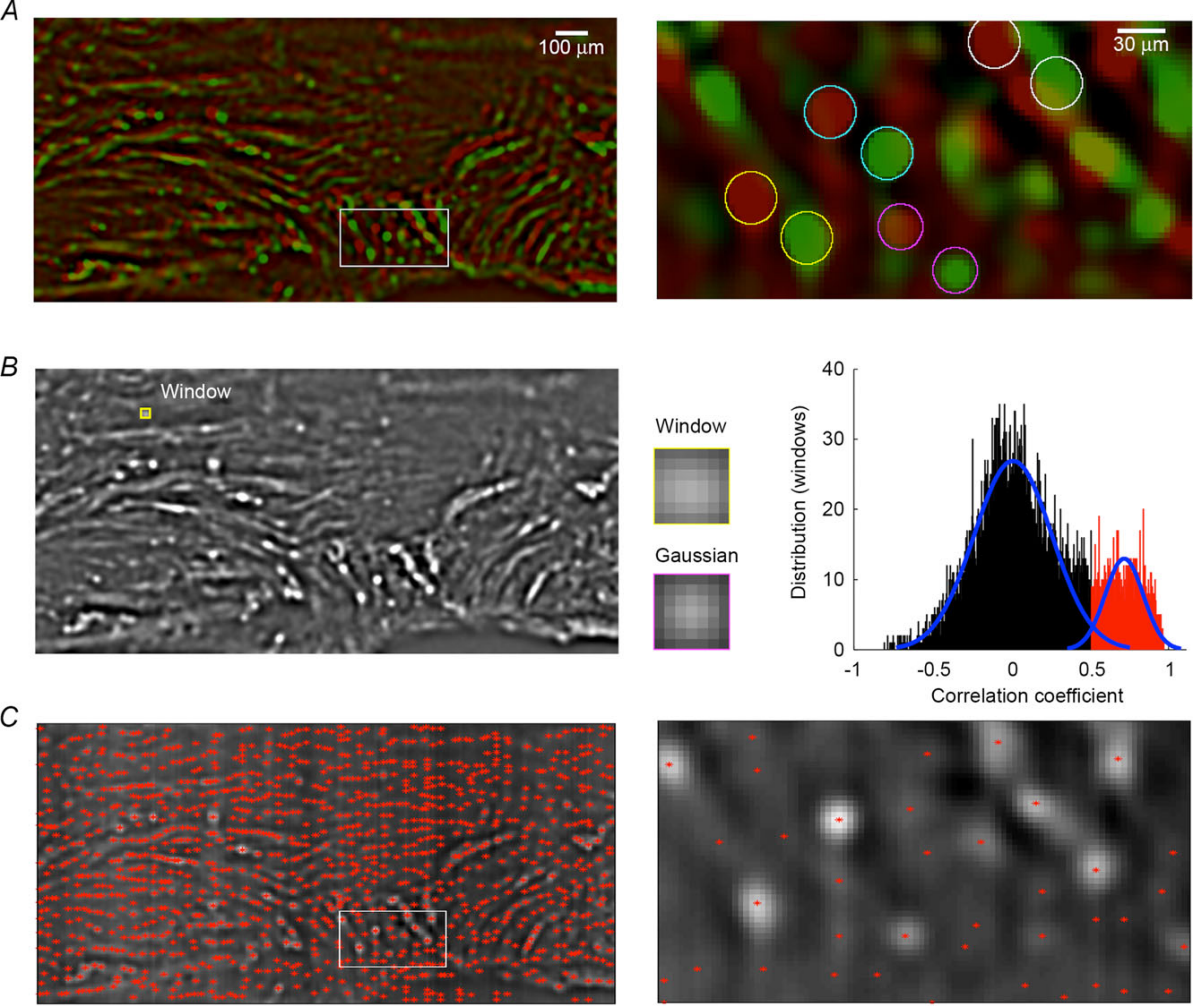
Identification of landmarks for tracking *A*, frames are bandpass filtered to emphasize small cell bodies (landmarks) with radii of ∼15 μm, which were present in all datasets. Two frames have been superimposed to illustrate the motion but constancy in the distribution of landmarks: in the first frame, the tissue is relaxed (red), whereas in the later frame there is a contraction (green). Four landmarks have been highlighted in colour to indicate their respective positions in the relaxed and contracted frames. *B*, the entire first frame is scanned by a window of pixel size (2*r* + 1) × (2*r* + 1), where *r* = 3. The quality of fit of each window to a 2-D Gaussian function (see insets) is then evaluated by their cross-correlation. The distribution of correlations for each window in the first frame comprises two components, one for windows not containing landmarks (black) and one for windows with landmarks (red). These latter windows are selected by fitting the total correlation distribution to two normal distributions (blue curves). *C*, the initial frame with landmarks identified (red*).

In all datasets, small cell bodies of ∼15 μm radius were present and displaced predictably with little change in shape over contraction–relaxation cycles, although their fluorescence intensity changed markedly (Fig. 2*A*). These landmarks were emphasized by bandpass filtering each frame to suppress high-frequency noise and structures with low spatial frequency. For the majority of our datasets, at a pixel width representing 3.4–4.5 μm, we found that the landmark-to-noise ratio was maximized when the parameters were set such that structures of 10–18 pixels in size were highlighted in the bandpass filter. The first bandpassed frame was then scanned in its entirety by a square window of size (2*r* + 1) × (2*r* + 1) pixels (with *r* chosen so that the window was large enough to contain one landmark only: typically *r* = 3 was sufficient). Each window was normalized such that the sum of all pixel intensities was equal to 1 and compared with a 2-D Gaussian function of the form


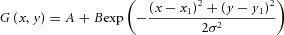
(1)

with parameters fixed (*A* = 0, *x*_1_ = *y*_1_ = *r* + 1, σ = (2*r* + 1)/3, *B* = a normalization constant set such that 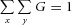). It was straightforward to identify windows containing landmarks by the quality of the correlation between the image contained within the window and the Gaussian function (see distribution in Fig. 2*B*). Windows enclosing landmarks had correlation coefficients close to 1 and those not enclosing landmarks had correlation coefficients close to 0, yielding a bimodal distribution of correlation coefficients. A two-component Gaussian mixture model was fitted to the correlation component distribution with the landmarks with higher correlation coefficients selected for tracking. The same landmark will be contained by a number of overlapping windows, but only the window with the landmark placed most centrally was retained. The average distance between the centre of a landmark and the centre of its nearest neighbouring landmark was 30 μm. After this last step, the positions *x*_1_, *y*_1_ of each landmark in frame 1 are known (Fig. 2*C*), as is the position in pixel coordinates of each (2*r* + 1) × (2*r* + 1) window centred on the landmarks.

#### Tracking landmarks between frames and removing outliers

The motion of landmarks between frames is relatively small and in the case of our data did not leave the enclosing windows from the previous frame. We therefore located the new landmark position in frame 2 by fitting the frame 2 data enclosed within the frame 1 window to the 2-D Gaussian function in eqn (1), but replacing *x*_1_, *y*_1_ with *x*_2_, *y*_2_ and optimizing parameters *A*, *B*, σ, *x*_2_ and *y*_2_ for the fit. Because the positions *x*, *y* are not restricted to pixel resolution, the centre of the Gaussian *x*_2_, *y*_2_ gives the fitted centre of the landmark at a sub-pixel resolution. Once this has been achieved, the position of the window is updated, if required. This procedure is repeated for all landmarks and for all subsequent frames to obtain the sub-pixel location of a landmark in each frame as a set of coordinates


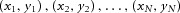
(2)

where *N* is the total number of frames over which the features are tracked. A number of criteria are applied to remove outliers and spurious tracks. If a landmark hits an image boundary, it is removed from the set of landmarks used for the later calculation of tissue-wide motion. Additionally, a four-stage filtering process is applied: (i) where landmark paths intersect, only that with the smoothest velocity in the periods before and after intersection is retained; (ii) landmarks with an average inter-frame distance that exceeds a threshold value are removed; (iii) landmarks with positions relative to neighbouring landmarks that significantly change are removed, and finally (iv) a low temporal resolution adaption of the local median test (Westerweel, 1994; Westerweel & Scarano, 2005) filters out the remaining outliers.

#### Characteristic length scale and motion-corrected images

Having obtained the trajectory of each landmark, we can estimate the movement of the tissue between landmarks; this is the final step in the motion-correction algorithm that leads to the data presented in Fig. 1*D*. This extrapolation is achieved through an optimal distance-weighted average of nearby landmark trajectories. Consider the displacement from the initial position in the first frame of a particular landmark, which we will label *l*


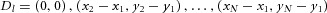
(3)

The displacement *D_p_* of the tissue under one of the pixels in the first image will be similar to that of nearby landmarks. If the displacement of only the nearest landmark is used to estimate *D_p_*, there will be discontinuity between displacements of adjacent pixels with different nearest landmarks. At the other extreme, if an unweighted average over all landmarks were to be used regardless of distance, all pixel displacements would be identical. There must therefore exist an optimal length scale, below which contracting tissue motion is locally homogeneous, for the estimation of *D_p_* from nearby landmark displacements. We will call this the ‘*characteristic length scale*’ for myometrium: although it is required for the motion correction, it is a key biophysical characteristic of the tissue structure because it gives the scale below which the tissue remains homogeneous under contraction. If we call ϕ*_pl_* the distance between the tissue under a pixel *p* and a landmark *l* in the first image frame, we can introduce the weighting function 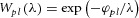, where λ is the length scale that must be optimized. We can write the distance-weighted estimate for the displacement *D_p_*(λ), for a particular guess of the scale λ, as


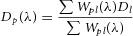
(4)

where the sums are over all tracked landmarks. It now remains to find the optimal value of λ. This is straightforwardly achieved by comparing the prediction of eqn (4) when the pixel *p* is a landmark itself (for which the displacement is known) for a range of different λ-values. The value which gives the best prediction of the known landmark displacements, using a weighted average of the other landmarks, we call the characteristic length λ*_c_* of myometrial tissue. Once found, the displacements given in eqn (4) are computed using the optimal λ*_c_*. To find the position in a subsequent frame of tissue that was under a particular pixel in the first frame, all we are required to do is to add its initial coordinates in the first frame to the displacement *D_p_*. Finally, the motion-corrected image sequence can now be constructed by mapping the intensity value found at a position in any later frame back to its point of origin in the first frame. As tissue positions are at sub-pixel resolution, bilinear interpolation is used to assign intensity value.

#### Output of algorithm

The motion-correction algorithm generates a sequence of tif files. These may then be analysed using any suitable image-processing software. To extract local intensity profiles, we imported the files into Fiji (Schindelin *et al*. 2012). Regions were identified in which the [Ca^2+^]_i_ levels either oscillated or remained constant between the global [Ca^2+^]_i_ transients associated with coordinated contractions. Regions of interest (ROIs) were selected to encompass regions of tissue with uniform [Ca^2+^]_i_ response and the Multi-Measure function was used to extract the mean intensity of each ROI over time.

### Local measures of contraction

Although the algorithm was principally designed to remove motion artefacts and facilitate the measurement of calcium transients, an informative byproduct of the method is that it provides a description of tissue kinematics during contraction at high spatiotemporal detail. This includes the changes in position, velocity and acceleration over time for each part of the tissue and can therefore be used to measure the strength of contractions locally. To this end, we calculated the change in the areas of tissue enclosed by a grid of points that were uniformly distributed in the first image frame. As the tissue contracts, the grid warps and the initially square regions enclosed by grid vertices deform, changing in size and shape. The coordinates of the corners of the deformed quadrilaterals were used to calculate the changing area contained for all frames, with the local area contraction Δ*K* defined as:


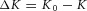
(5)

where *K*_0_ is the area of the square in the first frame and *K* is the area of the quadrilateral region as a function of time.

## Results

### Significant reduction of motion artefacts

**Figure 3:**
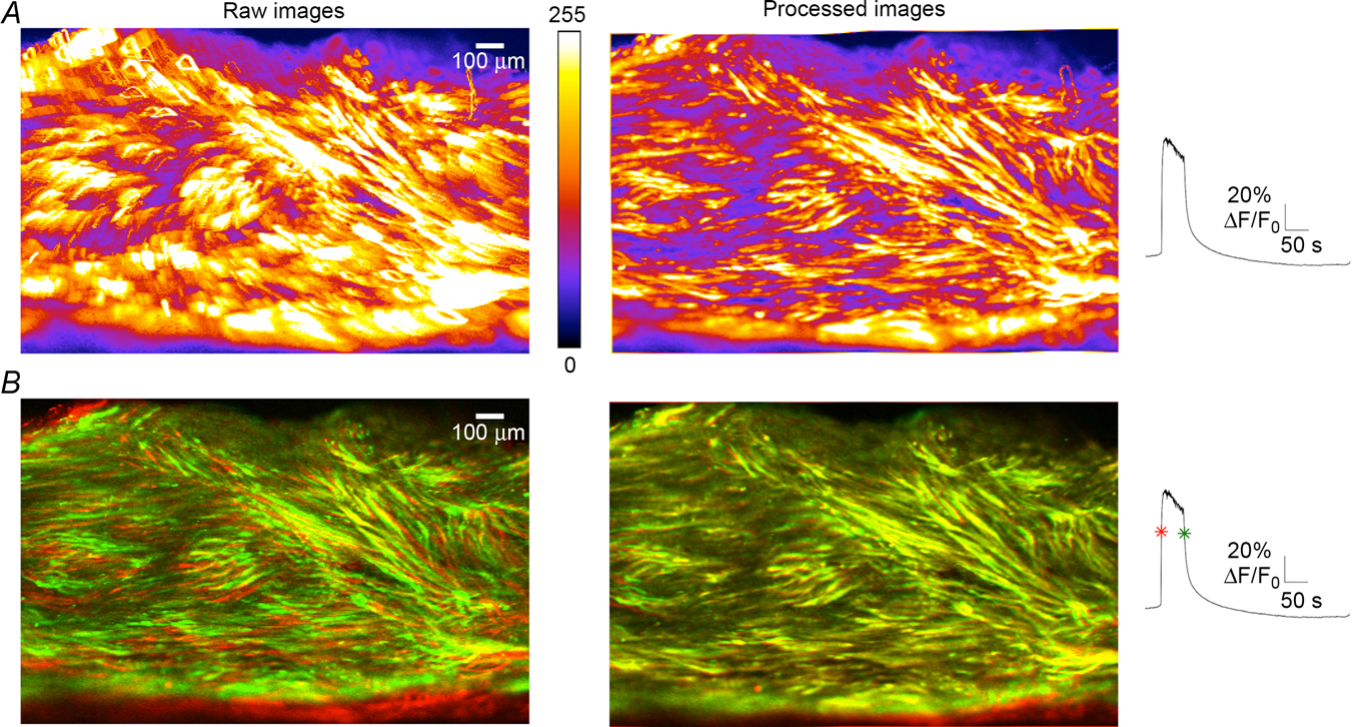
Elimination of motion artefacts in images of contracting myometrium *A*, maximum-intensity projection of the image sequence over a single contraction–relaxation cycle was calculated for raw (left panel) and processed (right panel) data. The trajectories of high-intensity regions in the raw images are significantly reduced in the processed images. The tissue-averaged change in baseline fluorescence (Δ*F*) over the contraction–relaxation cycle used in the maximum-intensity projection calculation is shown to the right of the two panels. *B*, raw fluorescence images (left panel) and images processed for movement reduction (right panel) showing the Fluo-4 loaded tissue slice in a contracted state (green) superimposed upon the imaged slice in a relaxed state (red). Improved alignment between the two frames can be seen in the processed images compared with the raw images. The positions of the two frames in the contraction–relaxation cycle are indicated in the tissue-averaged Δ*F* time course (far right, red and green*).

Our algorithm processes calcium indicator fluorescence imaging sequences of contracting myometrium to remove motion artefacts by tracking the movement of identifiable landmarks and extrapolating the data to obtain a complete description of the tissue-wide motion. This description is then used to produce a new ‘static’ image sequence from which contractile motion has been removed. The algorithm was tested in 15 datasets of imaged myometrium. In all of the datasets tested, processing the images resulted in a significant, if not complete, reduction of the motion artefacts over multiple contraction–relaxation cycles and across the entire imaged slice. Figure 3 illustrates the reduction of motion in one spontaneously contracting imaged myometrial slice (see also Supporting Movie 1). In Fig. 3*A*, the maximum intensity projections (each pixel takes its maximum value over the entire image sequence) for a single contraction–relaxation cycle are presented for raw and processed data. In the raw dataset, the trajectories of regions of high [Ca^2+^]_i_ intensity are clearly evident as they move into areas previously occupied by the lower-intensity intercellular matrix (Fig. 3*A*, left panel). After processing, the effects of motion are significantly reduced across all areas of the image (Fig. 3*A*, right panel). The tissue-averaged change in baseline fluorescence (Δ*F*) over the contraction–relaxation cycle used in the maximum-intensity projection calculation is shown to the right of the top two panels. In Fig. 3*B*, a single frame of the imaged tissue in a contracted state (green) is superimposed upon a single frame of the tissue in a relaxed state (red) for both the raw (left panel) and processed (right panel) datasets. The positions of the two frames in the contraction–relaxation cycle are indicated in the tissue-averaged Δ*F* signal (right, red and green*). A significant improvement in the alignment of the two frames of imaged tissue can be seen in the processed data compared with the raw data. Consistent throughout all datasets was the ability of the algorithm to reliably reduce motion artefacts in all regions of the image in which the tissue was intact and in focus. A lower quality of correction can be seen towards the bottom of the processed superimposed images in Fig. 3*B*, where the edge of the imaged tissue has curled over and so was out of focus. The algorithm was applied to datasets during both periods of significant motion – tissue contraction and relaxation – and periods of relative quiescence. When there is no motion, processing the images with our algorithm has no significant effect on the data. Experiments were performed using the myosin light chain inhibitors wortmannin and ML-7 as controls in order to further test whether the processing algorithm has any effect on [Ca^2+^]_i_ measurements uncomplicated by movement. However, at concentrations sufficiently high to inhibit contractions (>5 μm for wortmannin and ∼3 μm for ML-7), these inhibitors substantially affected [Ca^2+^]_i_ dynamics and would thus alter any [Ca^2+^]_i_ signal measured from these datasets after motion correction.

### Increased spatiotemporal information

**Figure 4:**
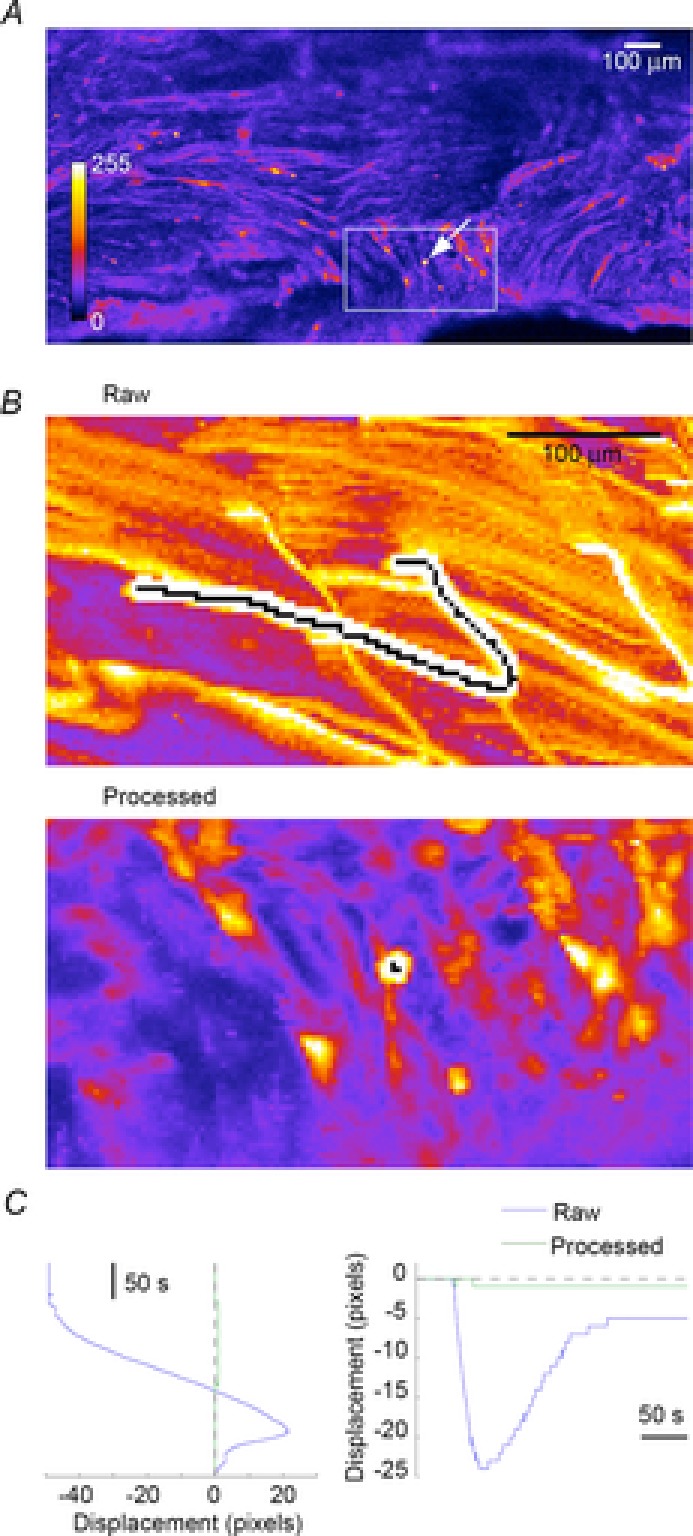
Improvement in effective spatial resolution down to pixel scale *A*, a feature of high intensity is identified (arrow) in the raw fluorescence image of a Fluo-4 loaded myometrial slice. *B*, the centre of the feature in (*A*) was tracked over a contraction–relaxation cycle in both the raw (top panel) and processed (bottom panel) images. Tracks are shown (black lines) on the maximum intensity projections of each image stack over the contraction–relaxation cycle. *C*, horizontal (left) and vertical (right) displacements of the feature centre for raw and processed data. Total displacement is reduced to a single pixel in both horizontal and vertical directions in the processed images.

Image sequences processed for motion reduction acquire an improvement in the effective spatial resolution of measurable calcium activity: the ability to measure from two adjacent points over the time series is increased. To quantify the improvement in effective spatial resolution afforded by our algorithm, we tracked the centre of a high-fluorescence feature over a contraction–relaxation cycle in both raw and processed images (Fig. 4). The identified feature (Fig. 4*A*, arrow) of diameter ∼20 μm maintained a stable fluorescence level throughout the contraction–relaxation cycle. In Fig. 4*B*, the tracked centre of the feature is shown (black line) on a maximum-intensity projection of the raw (top panel) and processed (bottom panel) images. The horizontal and vertical displacements of the centre of the feature were reduced from 71 pixels to 1 pixel and from 24 pixels to 1 pixel, respectively, following processing for motion reduction (Fig. 4*C*). In all datasets tested, processing the images for motion reduction resulted in an improvement in the effective spatial resolution down to pixel level (1.0–4.5 μm in our image sets).

**Figure 5:**
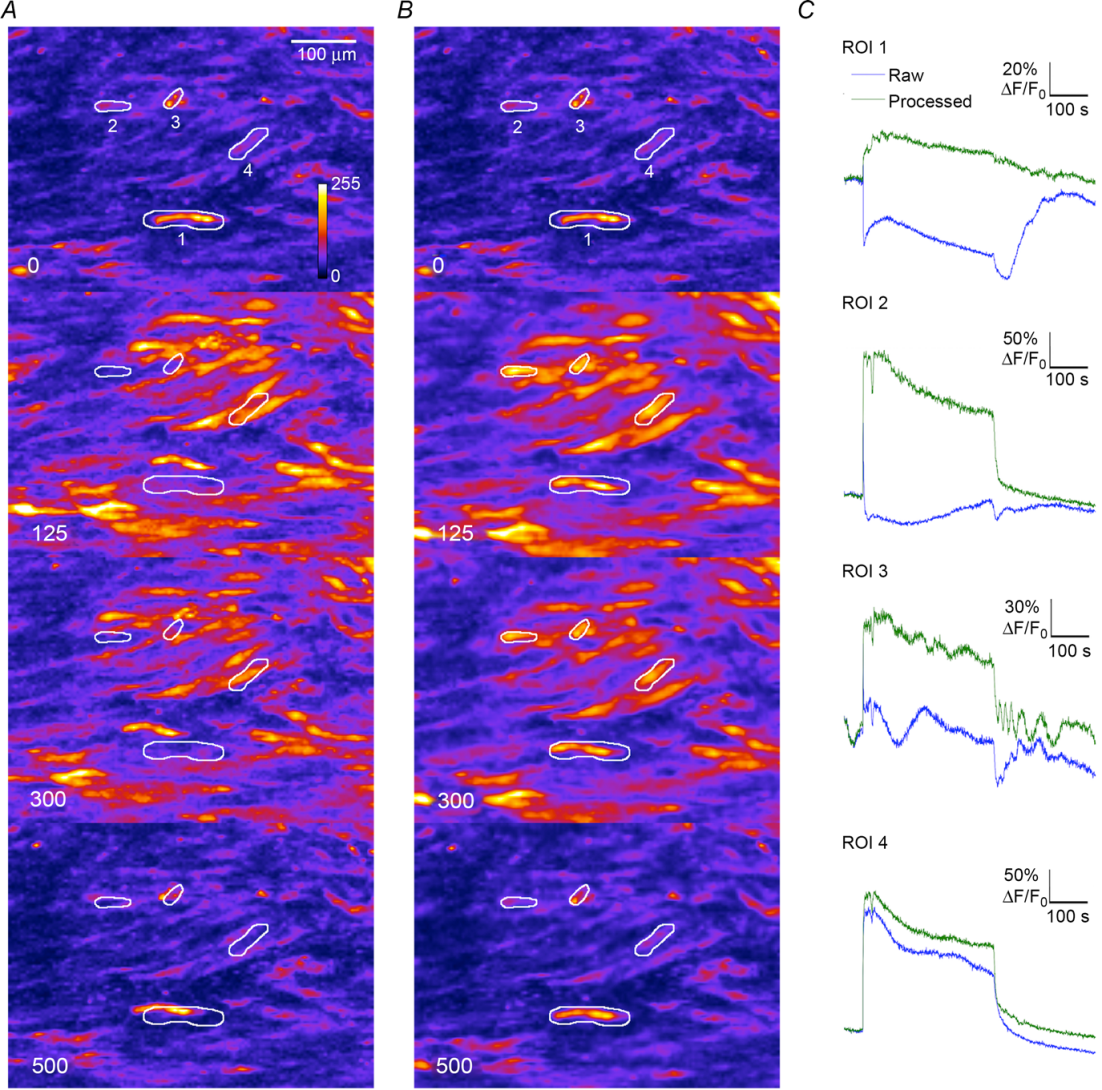
Motion correction permits easy detection of fine temporal detail in local fluorescence measurements *A*, *B*, magnified subsections of imaged tissue taken at times indicated (s) showing raw (*A*) and corrected (*B*) images over a contraction–relaxation cycle. Four regions of interest (ROIs) have been highlighted to illustrate the motion correction. *C*, time course of change in baseline fluorescence (Δ*F*) for the four ROIs for the raw and corrected images over the contraction–relaxation cycle.

The ∼50-fold improvement in effective spatial resolution allows simultaneous measurements of local changes in fluorescence to be taken from multiple regions of tissue. Figure 5 shows a magnified subsection of images of oxytocin-induced contracting tissue taken at four different time points over a single contraction–relaxation cycle for both raw (Fig. 5*A*) and processed (Fig. 5*B*) data. Four ROIs have been highlighted; the Δ*F* time course from within these ROIs is plotted in Fig. 5*C*. The ROIs were chosen to illustrate the increased information that can be obtained from motion-corrected images; the magnitude and kinetics of the traces will depend on the way in which the ROI is chosen and the indicator used (in this case Fluo-4), as well as on the underlying [Ca^2+^]_i_ activity. As is clearly evident from the panels in Fig. 5*A*, the Δ*F* time courses for the raw images reflect activity from changeable regions of tissue and cannot therefore provide information about [Ca^2+^]_i_ transients from within the regions of tissue highlighted in the top panel of Fig. 5*A*. Processing the images for motion correction allows the easy detection of fine temporal detail, as is particularly evident in the Δ*F* signal for ROI 3, where the region encompasses a microvessel with high-frequency fluctuations of [Ca^2+^]_i_.

### Spatial heterogeneity of [Ca^2+^]_i_ signals

**Figure 6:**
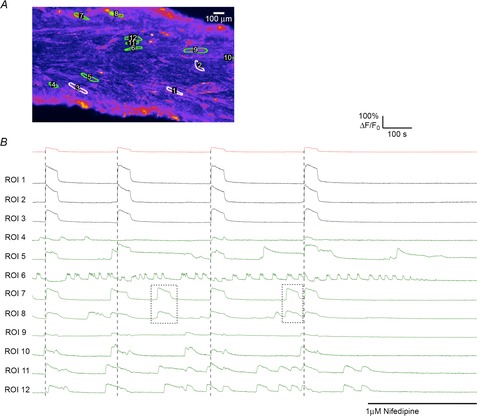
Spatial heterogeneity of [Ca^2+^]_i_ activity in contracting myometrium *A*, regions of interest (ROIs) encompassing sub-regions of tissue that exhibit periods between global contractions with either non-fluctuating (white) or fluctuating (green) [Ca^2+^]_i_ levels. *B*, change in baseline fluorescence (Δ*F*) over multiple contraction–relaxation cycles, averaged over entire tissue (red) and for the ROIs in (*A*) [non-fluctuating ROIs 1–3 (black), and fluctuating ROIs 4–12 (green)]. The onsets of peaks associated with global contractions are marked (vertical dashed lines). Note that the full contractile waveforms can be seen at intermediate times in a subset of the ROIs (7 and 8, dotted rectangles). Application of nifedipine resulted in the eventual cessation of global contractions and all heterogeneous activity.

In spontaneously contracting myometrium, cells in which [Ca^2+^]_i_ levels fluctuate between contractions have been identified in rat and human tissue (Burdyga *et al*. 2009; Bru-Mercier *et al*. 2012). Previously, direct comparisons of calcium activity within multiple cells in a contracting tissue slice was made difficult by the associated motion of the imaged tissue. Using our algorithm, we compared the transients of all sub-regions of tissue visually identified as exhibiting [Ca^2+^]_i_ fluctuations between contractions in a dataset in which the tissue was spontaneously contracting. Figure 6 shows the signals extracted from ROIs encompassing sub-regions that are quiet between contractions (ROIs 1–3) or show fluctuating [Ca^2+^]_i_ levels (ROIs 4–12). The Δ*F* signals reveal significant spatial heterogeneity of [Ca^2+^]_i_ activity between and during contractions. There are clear correlations in activity between some sub-regions that are not limited to the peaks associated with global contractions; for example, the similarity in the patterns of activity between ROI 11 and ROI 12 indicates a strong electrical connection between these two transversely neighbouring cells. The high spatiotemporal detail revealed in the motion-corrected data allows detection of local peaks in [Ca^2+^]_i_ activity similar in form to the peaks associated with tissue contraction occurring between global contractions: two such local peaks have been highlighted in Fig. 6 for ROIs 7 and 8. A noteworthy waveform is that of ROI 6, which exhibits high-frequency oscillations throughout the image sequence. These appear to be independent of the activity in other regions and may be indicative of the physiological behaviour of a myocyte in the cell cycle or a damaged cell. In Fig. 6, application of nifedipine resulted in the eventual cessation of all activity; however, this was not always the case. In a different tissue slice, application of nifedipine suppressed the Δ*F* peaks associated with global contractions, but not the fluctuations in Δ*F* occurring in some cells between contractions (see also Bru-Mercier *et al*. 2012). These results together suggest that Ca^2+^ entry through L-type channels is one, but not the only, mechanism underlying the asynchronous [Ca^2+^]_i_ activity in populations of cells between global contractions.

### Characteristic length scale

During myometrial contractions, cells structurally deform neighbouring tissue and are themselves affected by nearby contracting cells. The elastic properties, spatial organization and force produced by contracting myocytes set the characteristic length scale λ*_c_* below which tissue deformation during a contraction is locally homogeneous. As described in the Methods section, this length scale is identified during the extrapolation of landmark motion to that of the surrounding tissue.

**Figure 7:**
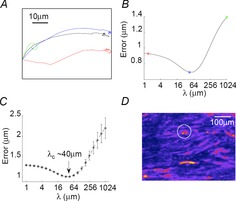
Characteristic length scale λ*_c_* of myometrium *A*, displacement of a landmark from Fig. 1 over 100 frames (black line) and distance-weighted estimate of displacement computed for λ = 2 μm, λ = 45 μm, λ = 1024 μm (red, blue, green lines, respectively). *B*, error for distance-weighted estimate computed for a range of λ-values, averaged over all landmarks in Fig. 1. *indicates values of λ used for plots in (*A*). *C*, normalized mean error per dataset for displacements of landmarks computed as the weighted average of all remaining landmark displacements for a variety of λ-values, with a minimum at λ*_c_*. *D*, visualization of contractile length scale (40 μm) centred on region of interest (ROI) 3 from Fig. 5.

For each dataset, the displacements of each landmark found by tracking were compared with a predicted displacement computed as a spatially weighted average of all remaining landmark displacements for a range of values of the spatial weighting parameter λ (see Methods). The optimal value of λ is that which minimizes the error between the displacements of each tracked landmark and the displacements computed as a weighted average across all datasets. In Fig. 7*A*, the displacement of a landmark from Fig. 1 over 100 frames is plotted (black line), along with its displacement computed as a weighted average for three values of λ (λ = 2 μm, λ = 45 μm, λ = 1024 μm; red, blue, green lines, respectively). The mean error for displacements of all landmarks in this dataset for a range of λ-values is shown in Fig. 7*B*, in which the values used for plots in Fig. 7*A* are indicated (*). In Fig. 7*C*, the mean total error per dataset for displacements of all landmarks is computed for each λ, revealing a minimum total error when λ =λ*_c_* *∼* 40 μm (results are the averages taken from 15 datasets). An illustration of the characteristic length scale is shown by the circle plotted in Fig. 7*D*, which is centred on a pixel located inside ROI 3 from Fig. 5.

### Kinematics of local tissue motion

**Figure 8:**
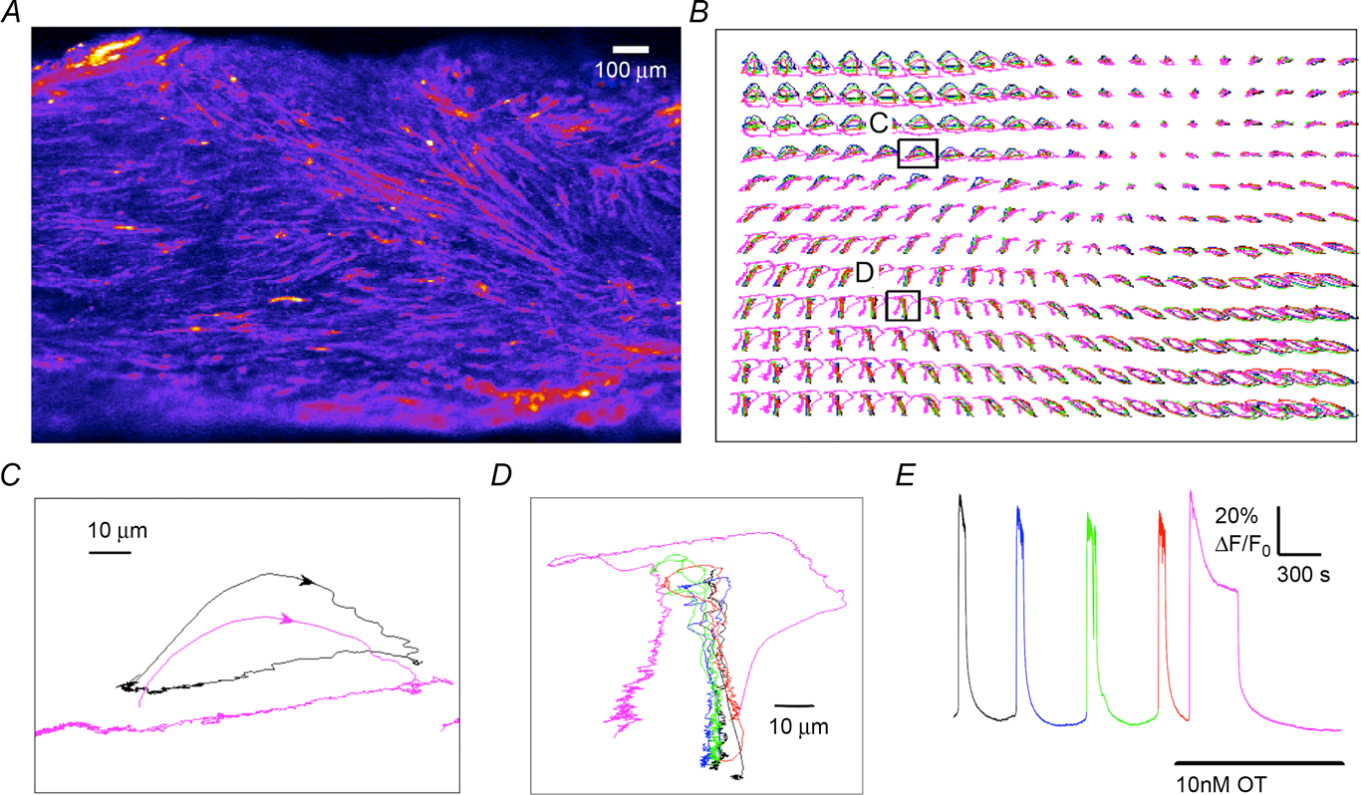
Kinematics of local tissue motion *A*, raw fluorescence image of a Fluo-4 loaded myometrial slice. *B*, paths of motion for evenly distributed points for five contraction–relaxation cycles. *C*, magnified subsection of (*B*) showing first and last contraction fields, with arrows indicating the direction of motion. Note that the trajectories are smoother during the contraction phase than in the relaxation phase. *D*, a second magnified subsection of (*B*) demonstrating path consistency across the first four spontaneous contractions with a wider path taken by the stronger oxytocin-induced contraction. *E*, time course of the fluorescence (Δ*F*) averaged over the entire tissue slice.

The description of local motion at all points within an imaged tissue slice, required for the motion-correction process, contains a wealth of information about the contractile properties of the tissue. We examined the kinematics of the tissue motion over multiple contraction–relaxation cycles for datasets in which the tissue was initially allowed to spontaneously contract for four cycles and was subsequently treated with 10 nm oxytocin. In Fig. 8*A* and *B*, the trajectories of initially evenly distributed points are shown over the course of five contraction–relaxation cycles. The associated Δ*F* signal averaged over the whole imaged slice is shown in Fig. 8*E* for reference. The tissue followed surprisingly stereotypical local paths in the spontaneous contractions (Fig. 8*B* and *D*, first four contraction–relaxation cycles). The application of oxytocin to the tissue in Fig. 8 caused a prolonged increase in [Ca^2+^]_i_ levels, which is reflected in protracted motion trajectories in some areas of the tissue (Fig. 8*B*–*D*). This reproducibility in spontaneous contractions was seen in three of four datasets examined. In the fourth dataset, the local contraction paths were similar for three spontaneous contractions; in the remaining cycle, a local contraction that did not propagate across the entire slice preceded the global contraction. The detailed measurements of local tissue trajectories that our method provides reflect the 2-D movement of the tissue slices comprising contractile motion and also motion artefacts resulting from the recording set-up, such as slippage of the tissue slice. However, the uniformity of motion seen in the majority of spontaneous contractions across datasets indicates a consistency in the underlying contractile mechanisms at both the cellular and network levels within the tissue slice. Of further note is the relative smoothness of motion of the tissue during the contraction phase in comparison with the subsequent relaxation phase (Fig. 8*C*). Analysis of the rough stochastic paths could potentially yield further information on the distribution and interaction of cellular mechanisms underlying muscle contraction and relaxation mechanics.

### Measuring local contraction

**Figure 9:**
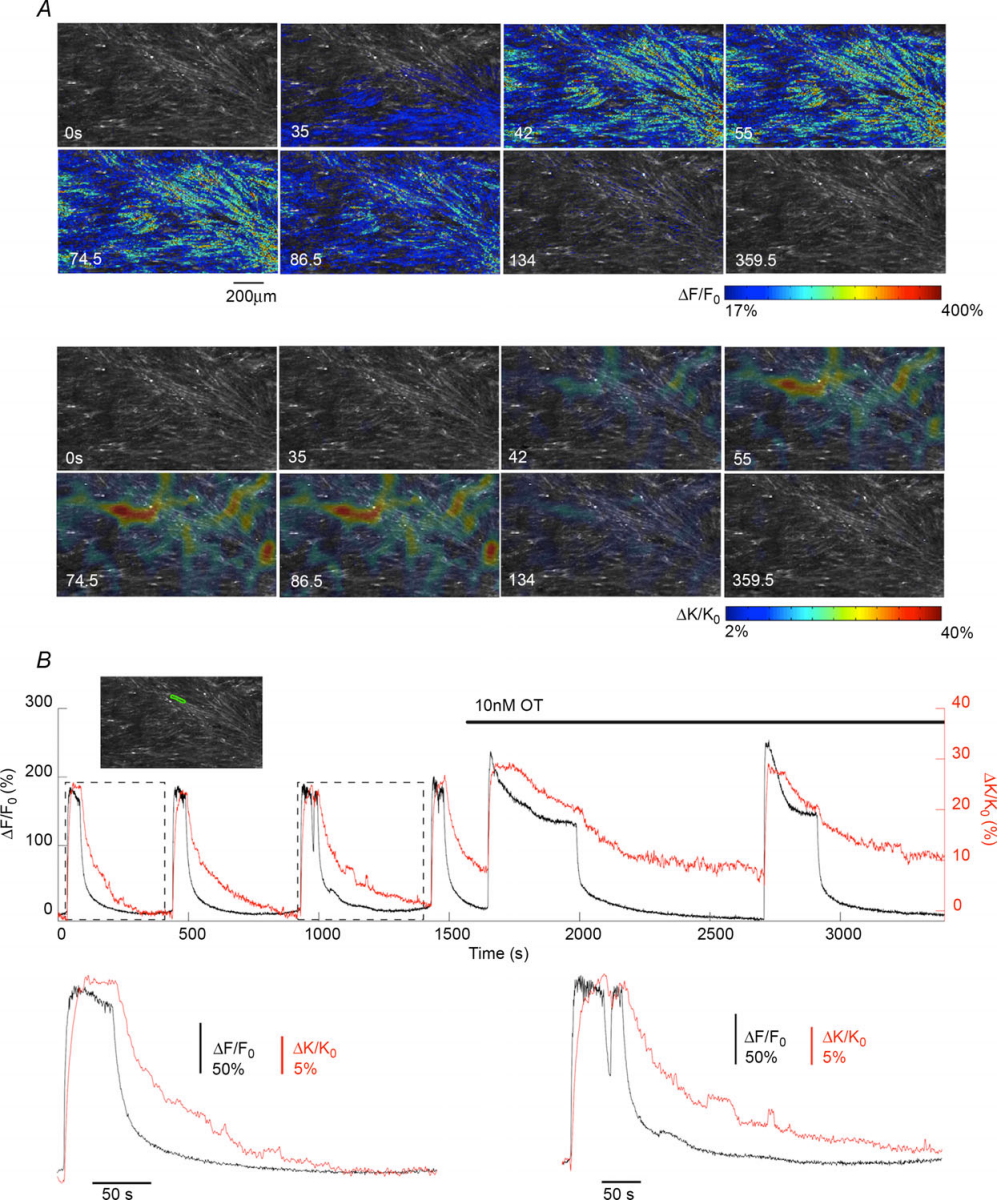
Comparison of [Ca^2+^]_i_ signals and spatiotemporal contraction field *A*, change in baseline fluorescence Δ*F/F*_0_ (top panels) and tissue contraction Δ*K/K*_0_ (bottom panels) for spontaneously contracting tissue over a single contraction–relaxation cycle. Changes in Δ*F/F*_0_ during contraction and relaxation are evident before changes in Δ*K/K*_0_ (see t = 35.0 s and t = 86.5 s, respectively). Spatial differences between regions of peak Δ*F/F*_0_ and Δ*K/K*_0_ levels highlight the dependence of the contractile properties of tissue regions on the surrounding structure. Changes shown for Δ*F/F*_0_ are ≥ 17% and Δ*K/K*_0_ are ≥ 2%, where thresholds are the means ± s.d. of data in the quiescent period preceding contraction. *B*, time courses of Δ*F/F*_0_ and Δ*K/K*_0_ over multiple contraction–relaxation cycles for a region of interest (ROI) (inset, green). Two contraction–relaxation cycles are magnified in the two bottom panels. The dynamics of Δ*F/F*_0_ transients occur at a faster time scale than those of Δ*K/K*_0_ transients (first contraction–relaxation cycle: 3 s for 20–80% Δ*F/F*_0_ during the rise compared with 9 s for Δ*K/K*_0_; 25 s for 80–20% Δ*F/F*_0_ during the fall compared with 88 s for Δ*K/K*_0_).

We used the kinematics of local motion to calculate the spatiotemporal structure of tissue contraction and compare this with local [Ca^2+^]_i_ dynamics. We calculated the contraction of each 10 × 10 μm region of tissue using the measure Δ*K* = *K*_0_ − *K*, where *K* is the deformed area and *K*_0_ the original area (*K*_0_ = 100 μm^2^). Figure 9 provides an example of a comparison of tissue contraction and [Ca^2+^]_i_ dynamics. Significant increases in Δ*F* (Fig. 9*A*, top panels) are evident just before the accompanying changes in Δ*K* (Fig. 9*A*, bottom panels). This is clearly seen for an ROI (Fig. 9*B*) in which the dynamics of the Δ*F*/*F*_0_ signal during the rise to peak occur at a faster time scale than the Δ*K*/*K*_0_ dynamics (first contraction–relaxation cycle: 3 s for 20–80% Δ*F*/*F*_0_ compared with 9 s for Δ*K*/*K*_0_). It can also be noted that the onset of decrease in Δ*F* is evident before Δ*K* decreases and furthermore that the rate of decay of Δ*K* is significantly slower than that of Δ*F* (Fig. 9*B*, first contraction–relaxation cycle: 25 s for 80–20% Δ*F/F*_0_ compared with 88 s for Δ*K/K*_0_). The complexity of the structure of myometrium and the role this plays in the contraction of the tissue is highlighted in Fig. 9*A*: whereas there is broad spatial correlation between changes in fluorescence and tissue contraction, the regions exhibiting the greatest change in fluorescence do not always match those with the greatest levels of contraction.

## Discussion

In this paper, we present an algorithm that tracks the motion of contracting myometrium during local [Ca^2+^]_i_ intensity changes. The imaged tissue slices used in this study contained multiple myocyte bundles separated by interstitial space and thus exhibited significant heterogeneity of movement. The method corrects motion artefacts down to the pixel level throughout the imaged slices, resulting in a 50-fold increase in effective spatial resolution. It therefore allows for a significant improvement in the quality of spatiotemporal information that can be extracted from contracting myometrium. We demonstrated how the method can be used to measure local changes in calcium indicator fluorescence, examine spatiotemporal correlations in these signals and identify heterogeneous behaviour within tissue, including inter-contraction oscillations and localized calcium increases in a sub-region of the myometrium that do not trigger global contractions. As well as analysing the calcium signal itself, the motion-correction algorithm provides a full description of the kinematics of motion: a characteristic length scale λ*_c_* ∼ 40 μm below which the tissue remains locally homogeneous during a contraction was identified and spatiotemporal patterns of contraction intensity and their time courses were extracted and compared with the concurrent local calcium signals. The method makes possible novel analyses of calcium indicator fluorescence imaging data in greatly improved spatiotemporal detail. In the hope that it will be useful to researchers analysing these and similar datasets, freely modifiable code written in the matlab environment has been provided in the supplementary material.

### Detailed spatiotemporal [Ca^2+^]_i_ activity

Tissue-averaged calcium indicator fluorescence and measurements of force during contraction–relaxation cycles can be used to investigate the effect of changing pharmacological conditions on excitation–contraction coupling (Word *et al*. 1994; Taggart *et al*. 1996; Longbottom *et al*. 2000; Noble & Wray, 2002; Jones *et al*. 2004; Fomin *et al*. 2006). However, understanding local heterogeneities in [Ca^2+^]_i_ activity may be key to elucidating the mechanisms governing the initiation of spontaneous contractions. Simultaneous measurements of local calcium indicator fluorescence transients taken from multiple regions of contracting tissue provide a way to characterize the spatial heterogeneity of [Ca^2+^]_i_ activity. Our method allowed us to measure calcium indicator fluorescence transients during and between contractions at the subcellular level, in which the choice of cells was not restricted by tissue motion (Fig. 6). Care must be taken when interpreting calcium indicator fluorescence imaging data for contracting tissue: changes in tissue thickness and the effect of spatial averaging contribute to the challenge of reliably inferring relative changes in [Ca^2+^]_i_ from fluorescence changes. These limitations are discussed in more detail below. However, our measurements allowed us to detect both correlated and uncorrelated fluctuations in intensity between peaks associated with global contractions in different tissue regions, which provide information about the electrical connectivity of the network. We found evidence that Ca^2+^ entry through L-type channels is one, but not the only, mechanism underlying the asynchronous fluctuations in [Ca^2+^]_i_ occurring in subpopulations of cells between global contractions (Fig. 6; see also Bru-Mercier *et al*. 2012). With the considerations about interpreting [Ca^2+^]_i_ activity from fluorescence measurements taken into account, our method will permit further investigation of locally asynchronous [Ca^2+^]_i_ activity.

### Kinematic and mechanical properties of tissue

The complete description of tissue-wide motion over contraction–relaxation cycles obtained as a result of motion tracking contains a wealth of information about the biophysics of the contracting tissue that can be exploited in force generation models. The close correlation between [Ca^2+^]_i_ activity and force measured in strips of myometrium (Taggart *et al*. 1996; Noble & Wray, 2002; Jones *et al*. 2004) has led to the use of [Ca^2+^]_i_ activity as a representation of force production, with measurement of both force production and [Ca^2+^]_i_ activity in these studies involving a tissue-wide spatial average. The description of tissue-wide motion combined with the fine spatiotemporal resolution fluorescence signals obtained as a result of our motion tracking means that, for the first time, it is possible to correlate local contraction and [Ca^2+^]_i_ activity (Fig. 9*A*). We saw spatial correlations between increases in calcium indicator fluorescence and tissue contraction, but found that regions exhibiting the greatest changes in fluorescence were not always associated with the greatest levels of contraction. This highlights the complexity of the structure of the myometrium and the role this plays in local contraction and force generation. High-resolution spatiotemporal information on [Ca^2+^]_i_ activity and tissue contraction will therefore be key to understanding the biophysics of contracting myometrium.

The application of the motion-correction algorithm revealed a characteristic length scale of myometrium, in the order of λ*_c_* ∼ 40 μm, below which tissue motion remains locally homogeneous: it is the length scale below which the tissue is relatively stiff (Fig. 7). Interestingly, this scale is less than the typical diameter of myocyte bundles of 100–300 μm (Young & Hession, 1999), which indicates that the tissue is significantly deformed during a contraction at the scale of bundles. Additionally, analysis of the local paths of tissue motion revealed a greater smoothness during the contraction phase: during the relaxation phase paths were significantly rougher and noisier (Fig. 8). These kinematic details provide information on the biophysical properties of contracting tissue and can be used to constrain mechanical models of myometrium.

We were able to characterize simultaneously measured time courses of fluorescence change and tissue contraction for ROIs over multiple contraction–relaxation cycles (Fig. 9*B*). The dynamics of the contraction–relaxation waveform, in both the rising and falling phases, were slower than the associated increase and decrease in fluorescence levels. These dynamics of local tissue contraction obtained by motion tracking allow for potential correlation analysis between structure and contraction in myometrium, which may provide information about the elastic properties of the constituent parts of the tissue.

### Comparison with other motion-tracking algorithms

Motion-tracking algorithms designed or adapted for biological images frequently rely on the assumption that local intensity remains constant throughout the image sequence. In images of contracting myometrium tissue, the problem of motion artefacts is confounded by the simultaneous local changes in intensity, which are both spatially and temporally heterogeneous. We compared our method with an established method that uses optical flow to detect motion (Lucas & Kanade, 1981; Baker & Matthews, 2004). Even after pre-processing the images to attempt to normalize the relative intensity across the sequence, this method significantly underperformed in comparison with that described here.

### Limitations and further applications

Our method for processing calcium indicator fluorescence images of contracting myometrial tissue significantly increases the amount of information that can be extracted. There are, however, limitations associated with extracting local information about [Ca^2+^]_i_ activity from calcium indicator fluorescence images, both processed and unprocessed; we address three of these limitations below. Firstly, the 2-D images contain information taken from a 3-D structure, and therefore signals extracted from surface myocytes may be contaminated with [Ca^2+^]_i_ activity from other cells within the third dimension of the tissue. This issue can be addressed by ensuring that any conclusions from calcium indicator fluorescence imaging data about [Ca^2+^]_i_ activity are drawn only after measurements have been taken from multiple cells and multiple datasets. In allowing multiple simultaneous local measurements to be taken, our motion-reduction algorithm assists with this. Secondly, the datasets used in this study were images of myometrial tissue loaded with the calcium indicator Fluo-4. Local intensity signals measured in calcium indicator fluorescence images of contracting tissue will be affected by changes in the thickness of the tissue associated with contractions and relaxations. This contributes to the challenge of interpreting changes in intensity in such datasets. Use of a ratiometric fluorescence probe, such as Fura-2, would minimize the effect of changeable tissue thickness. However, it is difficult, if not impossible, to use Fura-2 in ratiometric mode on the confocal microscope as a result of restrictions on the available laser lines for dye excitation. Our algorithm could equally well be used to process wide-field microscopy datasets; this may be a promising direction for the future of calcium indicator fluorescence imaging of contracting myometrial tissue. A drawback of wide-field microscopy, however, that would require attention is that image contrast is compromised by areas out of focus much more than in confocal microscopy. Finally, our algorithm allows calcium indicator fluorescence measurements to be taken from the same cell or subcellular compartment irrespective of its shift during contraction, but such measurements necessarily reflect a spatially averaged signal. Interpreting these signals can be complicated if the region contains a non-uniform distribution of Ca^2+^ ions, an issue that should be considered when inferring [Ca^2+^]_i_ activity from calcium indicator fluorescence intensity (Hyrc *et al*. 2007).

The algorithm identified small cell bodies in the slices and used these as landmarks for motion tracking. These small cell bodies were always present in the myometrial tissue slices – most likely invading leukocytes and/or damaged cells – and were ideal for tracking in low-magnification recordings of large areas. However, the algorithm could be applied to higher-magnification recordings for analysis of subcellular [Ca^2+^]_i_ dynamics: for example, use of an additional, calcium-independent dye, such as a nuclear stain or fluorescent beads, might provide the landmarks required for tracking. Simultaneous recordings from the two channels would then enable motion correction and the measurement of subcellular calcium indicator fluorescence. The algorithm has been designed for and tested in 2-D image sequences, but the methodology allows for a straightforward extension into three dimensions. The algorithm's potential to remove motion artefacts from other imaged biological tissue, such as in cardiac and neuronal images, is therefore not limited to 2-D data.

## Abbreviations

ROI: region of interest

## Supporting information

The following supporting information is available in the online version of this article.

Disclaimer: Supporting information has been peer-reviewed but not copyedited.

Supporting Movie 1. Illustration of motion-correction algorithm and extraction of kinematics of tissue motion.

Supporting Movie 2. Example calcium imaging myometrium dataset for use with matlab code.

Motion-correction code. Working example of matlab code for motion correction.
